# Huge variation in obtaining ethical permission for a non-interventional observational study in Europe

**DOI:** 10.1186/s12910-019-0373-y

**Published:** 2019-06-03

**Authors:** Dylan W. de Lange, Bertrand Guidet, Finn H. Andersen, Antonio Artigas, Guidio Bertolini, Rui Moreno, Steffen Christensen, Maurizio Cecconi, Christina Agvald-Ohman, Primoz Gradisek, Christian Jung, Brian J. Marsh, Sandra Oeyen, Bernardo Bollen Pinto, Wojciech Szczeklik, Ximena Watson, Tilemachos Zafeiridis, Hans Flaatten

**Affiliations:** 1 Department of Intensive Care Medicine, University Medical Center, University Utrecht, Heidelberglaan 100, 3584 CX Utrecht, The Netherlands; 2 Hôpitaux de Paris, Hôpital Saint-Antoine, Service de Réanimation Médicale, 75012 Paris, France; 3 Sorbonne Universités, UPMC Univ Paris 06, UMR_S 1136, Institut Pierre Louis d’Epidémiologie et de Santé Publique, 75013 Paris, France; 4 INSERM, UMR_S 1136, Institut Pierre Louis d’Epidémiologie et de Santé Publique, Paris, France; 5 Department of Anesthesia and Intensive Care, Møre and Romsdal Health Trust, Ålesund Hospital, Ålesund, Norway; 6 Department of Circulation and Imaging, Faculty of Medicine, NTNU, Norwegian University of Science and Technology, Trondheim, Norway; 7 Department of Intensive Care Medicine, CIBER Enfermedades Respiratorias, Corporacion Sanitaria Universitaria Parc Tauli, Autonomous University of Barcelona, Sabadell, Spain; 8 Laboratory of Clinical Epidemiology, GiViTI Coordinating Center, department of Public Health, IRCCS – “Mario Negri” Institute for Pharmacological Research, Ranica (Bergamo), Italy; 9 Unidade de Cuidados Intensivos Neurocríticos, Hospital de São José, Centro Hospitalar de Lisboa Central, Lisbon, Portugal; 10 Department of Anaesthesia and Intensive Care Medicine, Aarhus University Hospital, Aarhus, Denmark; 11 Department Anaesthesia and Intensive Care Units, IRCCS Istituto Clinico Humanitas, Humanitas University, Milan, Italy; 12 Department of Anaesthesiology and Intensive Care, Department of Clinical Intervention and Technology, Karolinska University Hospital, Huddinge, Sweden; 13 Clinical Department of Anaesthesiology and Intensive Therapy, University Medical Centre Ljubljana, Ljubljana, Slovenia; 14 Division of Cardiology, Pulmonology and Vascular Medicine, University Hospital Düsseldorf, Heinrich-Heine-University, Düsseldorf, Germany; 15 Department of Surgery, Mater Misericordiae University Hospital, Dublin, Ireland; 16 Department of Intensive Care Medicine, Ghent University Hospital, Ghent, Belgium; 17 Division of Anaesthesiology, Department of Anaesthesiology, Clinical Pharmacology and Intensive Care (APSI), Geneva University Hospitals, Geneva, Switzerland; 18 Division of Intensive Care and Perioperative Medicine, 2nd Department of Medicine, Jagiellonian University Medical College, Krakow, Poland; 19 Intensive Care, Kingston Hospital, Kingston, UK; 20 Intensive Care Unit, General Hospital of Larissa, Larissa, Greece; 21 Department of Anaesthesia, Haukeland University Hospital, University of Bergen, Bergen, Norway; 22 Intensive Care and Department of Clinical Medicine, Haukeland University Hospital, University of Bergen, Bergen, Norway

## Abstract

**Background:**

Ethical approval (EA) must be obtained before medical research can start. We describe the differences in EA for an pseudonymous, non-interventional, observational European study.

**Methods:**

Sixteen European national coordinators (NCs) of the international study on very old intensive care patients answered an online questionnaire concerning their experience getting EA.

**Results:**

*N* = 8/16 of the NCs could apply at one single national ethical committee (EC), while the others had to apply to various regional ECs and/or individual hospital institutional research boards (IRBs). The time between applying for EA and the first decision varied between 7 days and 300 days. In 9/16 informed consent from the patient was not deemed necessary; in 7/16 informed consent was required from the patient or relatives. The upload of coded data to a central database required additional information in 14/16. In 4/16 the NCs had to ask separate approval to keep a subject identification code list to de-pseudonymize the patients if questions would occur. Only 2/16 of the NCs agreed that informed consent was necessary for this observational study. Overall, 6/16 of the NCs were satisfied with the entire process and 8/16 were (very) unsatisfied. 11/16 would welcome a European central EC that would judge observational studies for all European countries.

**Discussion:**

Variations in the process and prolonged time needed to get EA for observational studies hampers inclusion of patients in some European countries. This might have a negative influence on the external validity. Further harmonization of ethical approval process across Europe is welcomed for low-risk observational studies.

**Conclusion:**

Getting ethical approval for low-risk, non-interventional, observational studies varies enormously across European countries.

## Background

When doing medical research there is a potential disparity between the interests of the researcher and the patient who is going to be subjected to the medical research. For this reason, medical ethical approval has to be obtained before research can be started. The medical ethical committees (ECs) or institutional review boards (IRBs) should weight the benefits of medical research against the potential harms for the patient. Interventional trials should only commence if, at least, the minimal legal, judicial and ethical standards are met. The European Union (EU) has issued a clinical trials directive (EU-CTD1) in 2001, which was implemented in the member states in 2004 [1]. Since then, more than 10 years have passed and a new clinical trials regulation has now been implemented (EU-CTD2) [2]. In this process, all countries have streamlined the process of getting approval for medical research and have appointed national medical ethical committees for the analyses and approval of medical research. This is especially well regulated for studies with an intervention, like (randomized) interventional trials.

However, observational research is generally considered as having a minimal risk for the participants. In many countries regulations have been developed to either exempt these studies from ethical review or provide the option of proportional review. These types of review are often delegated to the chair or smaller group of the ethical committee as they do not usually require a full board review [3]. However, up to date most of the research on timeliness of the ethical review has focused on those traditional interventional trials that require full review. We recently performed a multinational, observational study of very old critically ill patients (the VIP1-study) [4]. The goal of the VIP1-study was to analyze whether frailty was associated with poor outcome in this elderly patient group. Performing this research provided us with an opportunity to explore how differently ethical approval processes are working in various countries. The VIP1-study is prototypical for low-risk observational research. In short, in this study we included 5021 patients over 80 years old from 311 ICUs. We looked at reasons for admission, severity of disease, frailty, treatments during ICU (e.g. mechanical ventilation, renal replacement therapy, and vasopressor treatment), limitations in life sustaining therapy and outcome at 30 days. The data was collected in central online database and apart from age, gender and study number no other data was collected that could lead to identification of the patients. National Coordinators (NCs) were to apply for national ethical approval at the appropriate EC. We aimed for a waiver of informed consent but various countries demanded written informed consent prior to inclusion. For a more detailed description of the methods and definitions we refer to the publication of the VIP1-study [4].

Here, we describe the differences in the processes to obtain ethical clearance in the participating European and EU affiliated countries and the opinions of the National Coordinators about the medical ethical approval process.

## Methods

Originally, it was planned to get ethical approval from the participating countries in the first 6 months of 2016. The first center in Europe to apply for ethical approval for the VIP1-study started in May 2016. The last country started with this procedure in October 2016. Each country had a designated National Coordinator (NC), who was responsible for the process of getting national ethical approval. In February 2017 an online questionnaire concerning the experience of getting ethical approval with 11 questions was sent out to 16 European NCs (Table 1).

**Table 1:** The questionnaire to the national coordinators

	Question	Answer possibilities
Q1	Did you have to apply to more than one body in order to get approval for the study?	… yes … no … other, please specify
Q2	If Yes on Q1: Which bodies (committees) did you apply for consent?	… a national committee/body … a regional committee/body … a local hospital intitutional research board … other, please specify
Q3	How long was the application process from start to decision (weeks)?	…. weeks
Q4	Did you have to get informed consent for this study?	… yes, from the patient … yes, from the family … yes, but in retrospect from the patient … No, not deemed necessary
Q5	Is there a specific regulation when you send data electronically to a common database in another country?	… no … yes, it must be specified in detail on the application … yes, there is a seperate body and an independent application
Q6	Did you have to seek allowance to have your own (local) file/database with names of the included patients from your unit?	… yes … no … never thought of it
Q7	If YES from patient or family on Q4, how often was consent denied?	… never … occasionally … sometimes … frequently
Q8	Do you consider (personal opinion) it necessary to have informed consent for such a study?	… yes … no … uncertain … other, please specify
Q9	All in all: How satisfied are you with the ethical clearance process for this study?	… very satisfied … satisfied … neutral … unsatisfied … very unsatisfied … other, please specify
Q10	What is your opinion of having a common EU regulation on research ethics, and that acceptance in one country will bind the rest (similar to drug-approval regulations)	… I am in favour of this … I am not sure … I am against this … Other, please specify
Q11	The name of your country	…

### Definitions

Every country has its own procedure to judge medical research protocols. We defined a “national medical ethical committee” as a committee that has the authority to judge an application for the entire country. A “regional medical ethical committee” has the authority to judge a medical research protocol for a particular region or state but not for the entire country. In many countries a local hospital ethical committee has to judge the medical research protocol as well. Their judgement can only be used as approval for that particular institute. We defined them as “institutional research boards” (IRBs).

### Ethical clearance

The Regional Committee for Medical and Health Research Ethics, Western-Norway was asked to judge whether sending out a questionnaire on the ethical process to the NCs needed ethical committee review. They judged that sending out such a questionnaire was exempted from ethical review.

## Results

Half of the countries (*n* = 8/16) needed apply to only one national medical ethical committee (national EC), while the others had to apply to the national ECs and subsequently to the individual hospital institutional research boards (IRBs) (question 1).

The most frequent body to be approached (question 2) was a local hospital institution review board (IRB) (67%), followed by a regional EC (33%) and a national EC (25%). In one country all participating hospitals had to send individual applications to their respective IRBs since there was no national medical ethical committee. Most hospitals had to provide more specific information regarding the upload of data (question 5) to a central database abroad (*n* = 14/16). One country had to apply for this at a separate EC, because the national EC was not allowed to judge data collection in a foreign country. In *n* = 4/16 the NCs had to ask additional ethical approval to keep a local subject identification code list to de-pseudonymize the patients to be able to answer queries from the coordinating center (question 6). In 11 countries this was not necessary and in one country there was a separate application for this, resulting in two separate ethical approval rounds to the central EC.

**Fig. 1:**
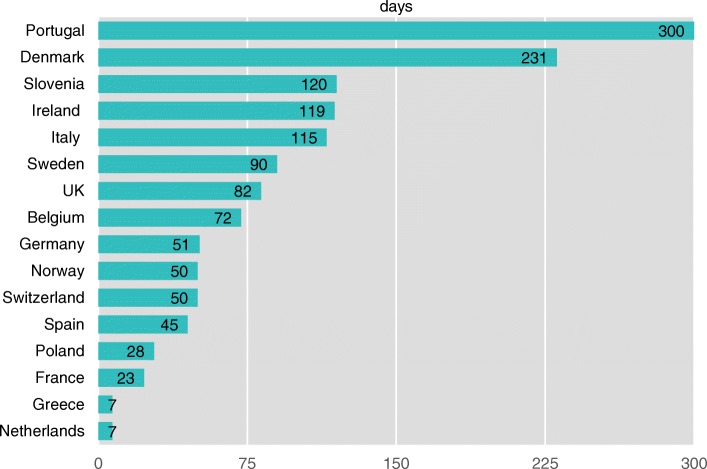
Time between application and first approval. Time between application and first approval to start or rejection from national Ethical Committee(s) or Institutional Research Board(s)

The time from application until the first national approval (question 3) is shown in Fig. 1. The time-to-approval ranged from less than a week to more than 300 days.

In *n* = 9/16 informed consent from the patient was not deemed necessary (question 4), and in *n* = 7/16 informed consent was prospectively required from the patient or the relatives or retrospectively from the patient. In 5 countries consent from the patients was necessary in advance, and in one country survivors had to consent in retrospect and only then was it allowed to use their data. One country allowed for consent by the family (proxy) without confronting the patient. Only *n* = 2/16 of the NCs agreed that informed consent was necessary for such an observational study (question 8).

**Fig. 2:**
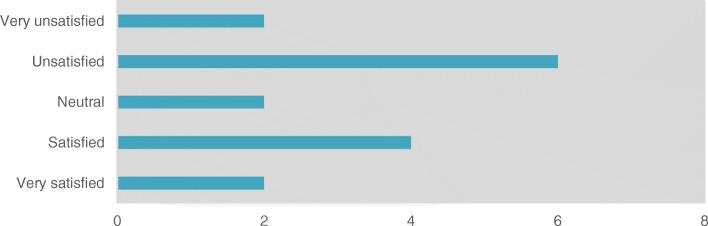
Overall satisfaction with the ethical approval rate. There was no obvious relation between being satisfied/very satisfied and the length of the approval process or a concordance between satisfaction and wanting centralized EU approval

Overall, *n* = 6/16 of the NCs were satisfied with the entire process of ethical approval, *n* = 8/16 were (very) unsatisfied with the process and n = 2/16 were neutral (time consuming but necessary) (question 9)(Fig. 2).

However, there was no clear correlation between the time-to-approval and being dissatisfied (*p* = 0.622). *N* = 11/16 would welcome a European central EC that would judge observational studies for all European countries, while *n* = 5/16 were “not sure” fearing “more bureaucracy” in the ethical approval process (question 10).

## Discussion

International observational studies of consecutive patients can reveal important differences in admission policies, treatments and outcomes of certain subgroups of ICU patients. Such observational studies often collect pseudo-anonimized or coded patient information and no particular treatment or intervention is imposed upon the subjects of the study. The potential harm to the subjects is, therefore, negligible and most would claim none. One could assume that these observational studies are assessed by the national ethical committees in an identical swift modus and the need for informed consent is waived. On the contrary, here we describe that this process went from smooth to such a prolonged period that one country could not even enter the study before the proposed end date [5].

In this survey, covering 16 European countries, we have demonstrated that there is very large variety of the ethical processes and outcome from either national ECs’, regional ECs’ or IRBs’ approval with regard to an identical study protocol. In most countries, more than one level of ethical approval had to be approached. Often a national EC needed to assess the research protocol and, once approved, the local IRBs needed to consent with the protocol as well. The time from applications to decision was unexpectedly large and only a few countries received feedback within a month from their national EC [6–8]. After a national EC has cleared the study local IRBs need to assess whether the study can be performed at their institution. Sometimes local IRBs are stricter than their national ECs and sometimes these IRBs needed additional information that was lacking from the original study protocol. Clearly, the differences in “approval time” are not solely the responsibility of the ethical committees. Sometimes insufficient information in the protocol is reason for additional queries. This will lead to additional delay and the majority of ICUs needed additional time between start of the national EC procedure and the final local IRB approval [9]. Moreover, after this study was performed most of the European countries that have participated in our research have implemented the new General Data Protection Regulation (GDPR) which has had a major impact on the collection of observational data [10].

Another major disparity between countries was the need for informed consent. A basic requisite for medical research is that every patient has the right to decide whether or not he/she will participate in medical research. However, if the patient is unable to consent because of severe illness informed consent could be asked retrospectively. However, some patients might not be able to give informed consent afterwards (because they are deceased or cognitively impaired). Leaving these patients out of the analyses might hamper the external validation of the research. In such cases the researchers can apply for a waiver of informed consent to the EC. The EC can grant such a waiver provided that the research poses important societal value and the research poses no more than minimal risk to the participants. We think this applies to observational studies in critically ill, elderly patients. The majority of countries were allowed to perform this study without any informed consent from the patient, but in a substantial amount of countries such permission had to be sought before inclusion of the patient [11]. If a patient is, for medical reasons, not able to consent (e.g. unconscious, sedated, etc.) and subsequently dies, this will create potential loss in patient recruitment subsequently leading to a selection bias and this may diminish the validity of the study [12]. A potential solution is to accept that survivors may claim their data to be removed (retrospective consent) but allow inclusion of data from deceased patients, despite the bias that also this option can introduce on the final results [13, 14]. However, only one country used this method and from the 226 included patients only one patient opted for removal of his data. The majority of national coordinators (*n* = 14/16) judged this observational study as having such a low potential of harm to the subjects that they would not need informed consent for this study. However, this leaves *n* = 2/16 that are convinced that consent is necessary even for anonymous observational registries.

Half of the national coordinators were not satisfied with the process. There was no obvious concordance between the time needed to get the study approved by the national EC and satisfaction. However, the countries in which the verdict was received swiftly were invariably satisfied with the entire process. Clearly a swift approval process leads to higher satisfaction. Especially in countries with a very prolonged EC process the national coordinators were dissatisfied and more willing to accept a centralized European ethical clearance entity for non-interventional studies/registries. At present, a new clinical trials directive (EU-CTD2) is being implemented. However, this directive particularly focuses on interventional trials (that, indeed, have a high potential risk of harm for the medical subjects). This leaves a gap in international legislation for pseudo-anonymous observational registries. The majority of the national coordinators would welcome a central European ethical organization for observational studies which would speed up national and local approvals. The general consensus was that such a non-interventional study should not take prolonged periods to be approved.

We have learned that we need to allow for up to one year to get all the countries to approve such a study [15, 16]. Not incorporating this “waiting time” in study protocols will lead to loss of countries and will, therefore, diminish the external validity of the findings for the European population. We presume more delay in approval for studies with a higher potential of harm for patients (like interventional studies or even patient questionnaires) [17, 18].

Obviously, this analysis has limitations. We only asked the national coordinators of the VIP1-study to provide us with their ethical approval experiences. After the national coordinators had gained ethical clearance many local researchers needed to go through a similar experience at their local IRBs. A swift approval at a national level may not be followed by a similarly swift approval at a local level, or vice versa. Such data were not incorporated in this analysis. Obviously, the results from our study are not necessarily transferrable to other studies (limited external validity).

## Conclusion

We conclude that further harmonization between European countries in ethical clearance for observational, non-interventional studies and registries is desirable. However, these differences between European countries need to be considered and incorporated in the research plan in order to prevent missing important contributors.

## Data Availability

The datasets used and/or analyzed during the current study are available from the corresponding author on reasonable request.
